# Identification and phylogenetic inferences on stocks of sharks affected by the fishing industry off the Northern coast of Brazil

**DOI:** 10.1590/S1415-47572009005000039

**Published:** 2009-05-01

**Authors:** Luis Fernando da Silva Rodrigues-Filho, Tainá Carreira da Rocha, Péricles Sena do Rêgo, Horacio Schneider, Iracilda Sampaio, Marcelo Vallinoto

**Affiliations:** 1 Laboratório de Genética e Biologia Molecular, Instituto de Estudos Costeiros, Universidade Federal do Pará, Bragança, PABrazil; 2 Universidade Estadual do Maranhão, São Luis, MABrazil; 3 Centro de Investigação em Biodiversidade e Recursos Genéticos, Universidade do Porto, VairãoPortugal

**Keywords:** sharks, molecular identification, phylogenetic, mtDNA, conservation

## Abstract

The ongoing decline in abundance and diversity of shark stocks, primarily due to uncontrolled fishery exploitation, is a worldwide problem. An additional problem for the development of conservation and management programmes is the identification of species diversity within a given area, given the morphological similarities among shark species, and the typical disembarkation of processed carcasses which are almost impossible to differentiate. The main aim of the present study was to identify those shark species being exploited off northern Brazil, by using the 12S-16S molecular marker. For this, DNA sequences were obtained from 122 specimens collected on the docks and the fish market in Bragança, in the Brazilian state of Pará. We identified at least 11 species. Three-quarters of the specimens collected were either *Carcharhinus porosus* or *Rhizoprionodon* sp, while a notable absence was the daggernose shark, *Isogomphodon oxyrhyncus*, previously one of the most common species in local catches. The study emphasises the value of molecular techniques for the identification of cryptic shark species, and the potential of the 12S-16S marker as a tool for phylogenetic inferences in a study of elasmobranchs.

## Introduction

The natural stocks of many shark species are in sharp decline in most parts of the World (Baum *et al.*, 2003). Studies of these elasmobranchs have shown that the most significant threat to this group is anthropogenic – primarily from fishery industries – resulting in a growing number of extinction-threatened species (Camhi *et al.*, 1998; Dulvy *et al.*, 2003). The effects induced by removing these predators from the ocean food web remain unpredictable. However, Myers *et al.* (2007) provide evidence of oceanic ecosystem transformations.

A decline of up to 89% has been recorded in the abundance of certain coastal species in the northwest Atlantic (Baum *et al.*, 2003). Baum and Myers (2004) have suggested that the downward trend in the abundance of many shark species, especially in the Gulf of Mexico, began with the onset of industrialised fisheries. They cite the example of the oceanic *Carcharhinus longimanus*, which, in the 1950's, was originally one of the most common species in the Gulf of Mexico, but has since declined by 99% to date. Shepherd and Myers (2005) found that the populations of 16 shark species are diminishing in the Gulf of Mexico, mainly as a result of incidental harvesting. However, reliable data on the exploitation of elasmobranch stocks are scarce, and it seems likely that the number of shark species being harvested by local sport and commercial fishermen is considerably underestimated (Cortés, 2002).

In Brazil, sharks are harvested intensively, primarily for the commercialisation of fins and secondarily, their meat. Between 1980 and 1994, these accounted for 6.4% and 12.7% of total fishery catches in the states of Paraná and Santa Catarina, respectively (Paiva, 1997). In Paraná, the contribution of this group to the total catch was larger than that of important teleost species, such as weakfish (5.9%) and mullet (5.1%). This demand has maintained annual catches of sharks in Brazil at around 30,000 tons over the past two decades, and has resulted in a number of species being classified as endangered (IBAMA, Instrução Normativa n. 5, May 21th, 2004).

In the Brazilian state of Pará, commercial catches of sharks are mainly disembarked in the ports of Belém, Bragança and Vigia, where they are most easily marketed (Fundação PROZEE, 2006). Annual shark catches in this state were 6750-7570 tons between 1997 and 2000, although productivity has fallen over recent years, the total reaching less than 4400 tons in 2004 (CEPNOR/IBAMA, 2004; Fundação PROZEE, 2006).

As in the Gulf of Mexico, an important aspect of the harvesting of sharks in this region – which encompasses the Amazon estuary – is the fact that these animals are captured primarily during fishing for other target species, such as Tuna (*Thunnus*), Spanish mackerel (*Scomberomorus brasiliensis*) and Red snapper (*Lutjanus purpureus*), and are thus normally harvested incidentally (Szpilman 2004; Elias MP, MSc Dissertation, Universidade Federal do Pará, Belém, PA, 2004). Given this, there are few reliable data on either the species of shark being harvested in this region or catch sizes, and thus both parameters are probably considerably underestimated.

Therefore, sharks are extremely vulnerable worldwide to fishing practices of almost all types and descriptions, the effects of this situation being further aggravated by a number of characteristics specific to the group, such as their slow growth and maturation rates, and low fecundity (Hoenig and Gruber, 1990).

On considering these factors, the Brazilian Environment Ministry has included a number of species on its red list (MMA, 2004), this including the sandbar shark (*Carcharhinus porosus*), lemon shark (*Negaprion brevirostris*), daggernose (*Isogomphodon oxyrhyncus*), nurse shark (*Ginglymostoma cirratum*), whale shark (*Rhincodon typus*), and all the species of the genus *Sphyrna*. However, scarce reliable data are available on the elasmobranch species being harvested or the size of catches (Lessa *et al.*, 1999), this being especially the case in northern Brazil, where research lags far behind growth of the fishery industry.

A wide knowledge of the fish species that occur within a given area and their relative importance for local fisheries, are vital for a working understanding of stock dynamics. Together with existing information on species traits, survey data on current population characteristics provide an essential baseline for the development of management plans at the species level. Management of fish stocks is amply recommended (FAO, 2000), but is generally hindered by a lack of species-specific data. This situation is aggravated in the case of sharks, due to morphological similarity among species, thus hampering reliable taxonomic identification (Stevens and Wayte, 1998; Coelho and Erzini, 2008; Valenzuela *et al.*, 2008). Worse still, most commercial fishermen process sharks at sea, removing the head, entrails and fins prior to disembarkation (Castro, 1993).

On considering these problems, the use of molecular tools for the identification of shark samples has grown considerably in recent years, this resulting in the development of diverse techniques based on RFLP (Heist and Gold, 1999), mitochondrial DNA sequences (Hoelzel, 2001; Douady *et al.*, 2003; Greig *et al.*, 2005; Iglésias *et al.*, 2005; Ward *et al.*, 2008) and species-specific primers or repeats (Pank *et al.*, 2001; Shivji *et al.* 2002, 2005; Abercrombrie *et al.*, 2005; Clarke *et al.*, 2006a; Magnussen *et al.*, 2007; Pinhal *et al.*, 2008). One of the most important studies on shark identification is that of Greig *et al.* (2005), based on the region of the mitochondrial genome which extends from the 12S rRNA gene to the 16S rRNA (12S-16S), and which has provided a phylogenetic signal adequate enough for the discrimination of at least 35 shark species. Using the same mitochondrial region, Iglésias *et al.* (2005) were able to identify a paraphyletic arrangement in the family Scyliorhinidae, re-emphasising the difficulties not only of classifying this group, but also of understanding its evolutionary history.

All told, the identification of those shark species being exploited by the fishery industry in northern Brazil will be fundamentally important for understanding the effects of harvesting patterns, and will provide an essential baseline for the development of conservation and management programmes. As in other regions, processing the catch at sea impedes reliable identification of species after disembarkation, making the use of molecular markers essential for efficient recognition of species and monitoring shark stocks. Given these considerations, the primary objective of the present study was to obtain species-specific DNA sequences from sharks disembarked by local fisheries in northern Brazil, through comparisons with data available in the literature (Greig *et al.*, 2005; Iglésias *et al.*, 2005).

**Figure 1:**
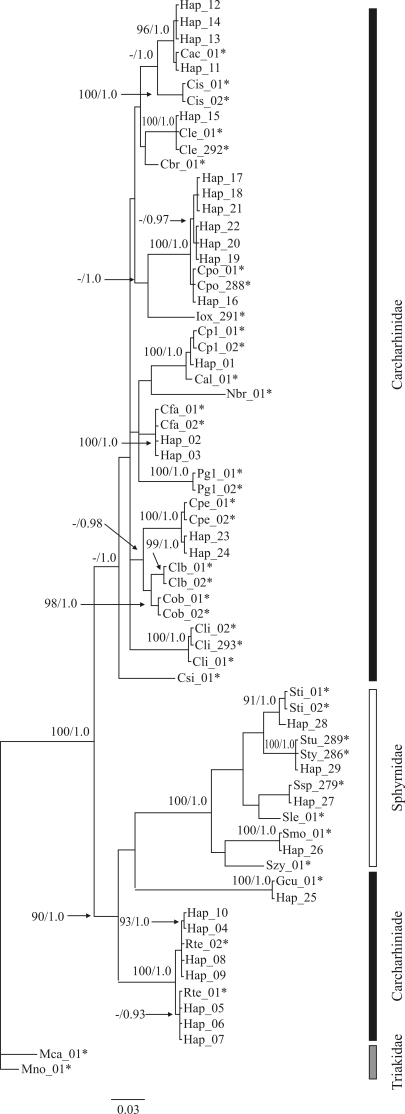
Phylogram based on a Bayesian analysis of mitochondrial DNA sequences (12S-16S), rooted with *Mustelus norrisi* and *Mustelus canis* (Triakidae). Support values are Bayesian posterior probabilities (right) and ML (left), nonparametric bootstrap values (1000 replicates) in percent. Asterisks indicate voucher specimens. (**-**) represents ML values below 90%.

## Materials and Methods

### Sampling, DNA extraction and sequencing

In the present study, 36 sequences available in the literature (Greig *et al.*, 2005) were obtained from GenBank (Table 1). Furthermore, seven individuals collected whole were identified by using the Compagno (1984) species key, the data therefrom being inserted into the data bank for comparison and possible identification of species (referred to as “present study” in Table 1). The species *Mustelus norrisi* and *Mustelus canis* (Triakidae) were used as out-group. For the identification study, 122 samples of shark tissue were collected from processed carcasses, known locally as *charutos* (from which the head, entrails and fins had been removed), at the municipal fish market in Bragança and at the docks in Bacuriteua and Bragança (in the Brazilian state of Pará) between October, 2005 and December, 2006. The samples were labelled according to the common name attributed to each individual by the fisherman or fishmonger from whom each specimen had been obtained, fixed in 95% ethanol and stored at -20 °C until DNA extraction.

For extraction, the tissue was dissolved by using a solution of SDS and proteinase K. Total DNA was isolated following the Sambrook *et al.* (1989) rapid phenol-chloroform extraction protocol, with precipitation by sodium acetate/isopropanol. Following extraction, a region of the mitochondrial genome (mtDNA) which stretches from the region of the 12S rRNA gene, passing through the tRNA-Valine gene to the 16S rRNA segment was amplified by PCR using the primers 12SA and 16SA (Greig *et al.*, 2005).

The PCR protocol was standardised to a final volume of 25 μL containing 0.25 μL of each primer (12SA = 5 pmol/μL, 16SA = 5 pmol/μL), 1 μL of MgCl_2_ (25 mM)_,_ 4 μL of dNTP mix (1.25 mM), 2.5 μL of 10x buffer (Invitrogen – Tris-HCl and KCl, pH 7.8), 0.2 μL (5 U/μL) *Taq* polymerase (Invitrogen), approximately 100 ng of total DNA and purified water to complete the final volume. The temperature cycles followed recommendations by Greig *et al.* (2005).

Following amplification, 2.5 μL of the product was purified using an Exo-SAP-IT kit (Amersham-Pharmacia) and sequenced by the dideoxytermination method (Sanger *et al.*, 1977), with reagents from the DYEnamicTM dye terminator kit (MEGABACE: Amersham Biosciences UK). Samples were sequenced in a MegaBACE 1000 (GE HealthCare) automatic sequencer. In addition to the primers mentioned above, the internal primer 12SINT was used to ensure complete sequencing of the target region (Greig *et al.*, 2005).

### Phylogenetic analyses

Sequences were edited and aligned using the CLUSTALW program (Thompson *et al.*, 1997) run in the BIOEDIT 5.0.6 package (Hall, 1999), with subsequent visual checking and manual correction of sequences.

The quality of the available phylogenetic information was evaluated following alignment and prior to data analysis. Saturation of substitutions was tested through a comparison of the number of transitions and transversions *versus* divergence, thereby providing a visual image of saturation through the DAMBE (Xia and Xie, 2001) program. This procedure was necessary due to the large number of taxa being evaluated, which included members from distinct families, and which could thus increase the probability of saturation among sequences.

The first step in the taxonomic identification of specimens was sequencing the seven known species, these then being added to the Greig *et al.* (2005) data base (Table 1). Inferences on the relationships between samples and the different taxonomic levels were based on uncorrected “p” distances. In this case, the aim was to determine the genetic distance (divergence) of the marker in question in order to define the limits between each taxonomic level.

For phylogenetic analyses, Maximum Likelihood (ML) trees were constructed using the GARLI program, version 0.951 (Zwickl D, PhD thesis, University of Texas at Austin, Texas, 2006). The preference for this program rather than the others available was due to its capacity to run large numbers of bootstrap replicates on large data bases, such as the one used here, which has both a wide variety of taxa and long sequences. The analysis was based on the General Time Reversible algorithm, and the significance of the groupings observed in all trees being estimated through bootstrap analysis based on 1000 pseudoreplicates. The robustness of this analysis was evaluated on considering bootstrap values of at least 90% as being statistically significant.

Bayesian analysis was carried out in the MrBayes programme (Ronquist and Huelsenbeck, 2003). This procedure is similar to ML, but differs in its approach to the use of probabilities. In Bayesian analysis, inferences are based on *a posteriori* probabilities of the phylogenetic trees (Schneider, 2007), which in MrBayes are estimated using Markov Chain analysis. We used four default heated chains, each of five million generations sampled every 100 generations by applying the stop rule command. The runs were subsequently evaluated for cut-off by using the Tracer program (Rambaut and Drummond, 2004).

## Results

A sequence of 1380 base-pairs was obtained for the 12S-16S region in 122 shark samples. Of these, 378 sites were variable and 325 were informative for parsimony analysis. Mean nucleotide composition was 25.5% Thymine, 22.0% Cytosine, 36.0% Adenine and 16.5% Guanine. Based on plotting divergence levels against transition and transversion rates, no evidence of saturation was found (not shown).

Bayesian and ML trees were constructed for the order Carchariniformes, as all the species identified in the study belonged to this group. The 122 sequences resulted in the identification of 29 different haplotypes (Table 2), which were used in phylogenetic analysis. The trees indicated that the samples belonged to at least 11 species (Figure 1), four of which from the hammerhead family Sphyrnidae (*Sphyrna mokarran*, *Sphyrna tudes,Sphyrna tiburo* and *Sphyrna* sp.). An un-identified species was closely aligned with *Sphyrna lewini* obtained from GenBank. A fifth species, the tiger shark (*Galeocerdo cuvier*), formed the sister group of the Sphyrnidae, distinct from the family Carcharhinidae.

The genus *Rhizoprionodon* (Carcharhinidae) was well-represented, with 35 individuals, divided into two well-defined subgroups in the trees. This genus was the sister group to the Sphyrnidae/*G. cuvier* dichotomy.

The genus *Carcharhinus* revealed the largest number of species, with five, namely *Carcharhinus falciformis*, *Carcharhinus leucas*, *Carcharhinus perezi*, *Carcharhinus acronotus*, and *Carcharhinus porosus*. The latter was the most common, with 57 individuals. A trichotomy was observed between the individuals of this group represented by the haplotype Hap_01 and sequences of *Carcharhinus plumbeus* and *Carcharhinus altimus* obtained from GenBank (Figure 1).

The genetic divergence values (uncorrected “p” distance) varied between 0.00% and 15.35% (Table 3). Inter-specific divergence between members of the Carcharhinidae and Sphyrnidae was between 7.66% and 10.00%. Similar values were recorded when comparing these two families with the Triakidae (6.65-9.80% for the Carcharhinidae, and 9.60-10.80% for the Sphyrnidae). Distances between *Galeocerdo* and other genera of the Carcharhinidae (7.56-8.63%) were similar to those found between families, whereas very much lower values (3.80-4.82%) were recorded between two other carcharhinid genera, *Carcharhinus* and *Isogomphodon*. The value for trichotomy involving *C. altimus*, *C. plumbeus* and Hap_01 was extremely low (0.53%), similar to intra-specific levels of divergence (see below), thus impeding reliable identification of specimens.

Regarding intra-specific divergence and despite the lack of a clear definition of divergence criteria for the differentiation of genera, the 12S-16S region proved to be effective for identifying species. All those specimens which diverged from identified species at the 0.0-0.4% level were allocated to the respective species, given that such values are well below the levels observed between distinct species (Table 3).

The identification of specimens by fishermen and fishmongers bore little relationship to their taxonomic classification (Table 4). Whenever a vernacular name was given to two or more specimens, they invariably represented at least two different genera. Similarly, only one identified species (*C. altimus*/*C. plumbeus*) represented by more than one specimen was consistently allocated to a single name - “Sacurí” - although sharks of five other species were also identified by this same name. Even the most commonly-used name (“Milho Verde”) was applied to a large number of individuals from two different genera. This quite emphatically confirms that the personnel involved directly in the exploitation of stocks have little reliable knowledge of the shark species involved.

## Discussion

Stevens *et al.* (2000) and Dulvy *et al.* (2003) have suggested that current trends of fishery exploitation of stocks of elasmobranchs are unsustainable, and that many shark species present a serious extinction risk. Some species, such as the blue shark (*Prionace glauca*) exceed maximum sustainable yield levels as a consequence of current trade volumes (Clarke *et al.*, 2006b). A major problem for the investigation and management of stocks is the identification of those species being harvested, especially difficult in the case of sharks, given both the taxonomic complexity of this group and typical processing of the catch at sea, when most of the diagnostic traits of the specimen are removed.

A number of studies – including the present one – have now shown that molecular markers can constitute an extremely effective tool for the resolution of taxonomic questions in shark species. Pinhal *et al.* (2008) used fragment-sizes from a PCR of 5S region repeats to identify different species of sharks collected in Brazilian and Venezuelan coastal sites. This was effective, but cannot be used for phylogenetic analysis.

The mitochondrial region used here permitted the identification of all the specimens collected, as well as contributing to the understanding of certain phylogenetic questions, thereby corroborating the results of Greig *et al.* (2005). It is important to note that intra-specific variation was low, this emphasising the monophyletic pattern of individuals from a given species. These same authors also noted that the low levels of intra-specific variability did not result in paraphyletic relationships among species. The same was observed here, with only one unsolved question: the arrangement between *C. altimus, C. plumbeus* and individuals represented by the Hap_01 haplotype. This may be due to paraphyly among these species, as observed by Greig *et al.* (2005) and Heist and Gold (1999) in their RFLP analyses of the cytochrome b gene, in which they found more substitutions between the Atlantic and Pacific populations of *C. plumbeus* than between the Atlantic populations of this same species and *C. altimus*.

In the hammerhead group, in addition to the three species identified unequivocally (*S. mokarran*, *S. tudes*, and *S. tiburo*), a distinct clade was observed, which was the closest to, but nevertheless well distinguished (divergence of 2.5%) from, *S. lewini*, and almost certainly represents a distinct but as yet unidentified species. One possibility is that this species is *Sphyrna media*, which is known to occur in the region, but was not represented in the data bank. It is also possible that two cryptic lineages of *S. lewini* coexist in the Atlantic (Quattro *et al.*, 2006). The resolution of these relationships is of major importance, considering that all the hammerhead species are considered to be especially vulnerable to the effects of commercial fishing (MMA, 2004).

It was not possible to reliably identify the different species of the genus *Rhizoprionodon*, given that the divergence recorded between the different subgroups (< 1%) was insufficient to arrive at reliable conclusions as to species status. The lack of data for other species of this genus - *e.g.Rhizoprionodon lalandei* and *Rhizoprionodon porosus* (Compagno, 1984; Lessa and Santana, 1998) - prohibits confirmation of the taxa recorded here. The occurrence of these two species off northern Brazil might nevertheless account for the two subgroups observed in the arrangement presented here (Figure 1).

One especially interesting result arising from the present study was the high divergence values observed between *Galeocerdo cuvier* and all the other carcharhinid species, which appears to contradict its inclusion in this group, thereby supporting Compagno (1984) and Szpilman (2004). With *Rhizoprionodon* as the sister group to a *Galeocerdo*/Sphyrnidae clade, the sum of evidence from analyses appears to indicate that both *Rhizoprionodon* and *Galeocerdo* may, in fact, represent distinct families. Certainly, the divergence values recorded for these two genera were consistent with those observed between members of the Sphyrnidae and Carcharhinidae, although this hypothesis would need to be tested with additional markers and analyses.

By contrast, the results of the present study also indicate that one of the most endangered species of the region – the daggernose shark, *Isogomphodonoxyrhyncus* - does in fact belong to the genus *Carcharhinus*, as argued by Compagno (1984). This species, once among the most common sharks on the Bragança fish market, was not even collected in our study. This further supports the classification of this species as critically endangered (MMA, 2004; IUCN), due to a combination of restricted geographic range and overexploitation by the fishery industry.

The *Carcharhinus* species identified in the present analysis are all known to occur in the study area (Compagno, 1984; Lessa *et al.*, 1999; Szpilman, 2004). Of these, *C. porosus* was by far the most common in our sample, as observed in previous studies (Elias MP, MSc Dissertation, Universidade Federal do Pará, Belém-PA, 2004). While this species is not listed internationally (IUCN), it is among those sharks considered to be endangered by the Brazilian government (MMA, 2004).

Summing up, the results of the present study not only confirmed the efficiency of the 12S-16S marker for the identification of shark species, but also emphasise its potential as a phylogenetic tool. Nevertheless, the arrangements presented here need to be tested with additional markers, considering that most of the groupings – especially those involving the Carcharhinidae – were not statistically significant. This is most likely related to the low levels of genetic variability intrinsic to sharks, which implies that the complementary analysis of more variable markers, such as the mitochondrial control region (D-loop), COI or the cytochrome b gene, would provide more definitive answers.

This is the first molecular study of sharks from northern Brazil. It was very successful in the taxonomic identification of cryptic specimens, especially those of the genera *Carcharhinus* and *Sphyrna*, which are normally classified to no more than the genus level (Ward, 2000; Chan *et al.*, 2003; Ward *et al.*, 2008). Thus, the molecular monitoring of local catches may prove to be an essential tool in the development of effective strategies for the conservation and management of shark populations in this region.

## Figures and Tables

**Table 1:** Reference species for the sequence identification recorded in this study. These include samples obtained from GenBank, and voucher specimens collected during the present study.

Family	Species	Code	N	English common name^a^	Local common name^b^	Source^c^
Sphyrnidae	*Sphyrna zygaena*	Szy_01	1	Smooth hammerhead	-	AY830772
	*Sphyrna lewini*	Sle_01	1	Scalloped hammerhead	*Pana; cornuda; martelo*	AY830768
	*Sphyrna* sp.	Ssp_279	1	-	-	Present study FJ598659
	*Sphyrna tiburo*	Sti_01; 02	2	Bonnethead	*Cação siri; bejoca; martelo*	AY830770-71
	*Sphyrna tudes*	Stu_286; 289	2	Smalleye hammerhead	*Cação siri; bejoca; martelo*	Present study FJ598662-63
	*Sphyrna mokarran*	Smo_01	1	Great hammerhead	*Pana; cornuda; martelo*	AY830769

Carcharhinidae	*Rhizoprionodon terraenovae*	Rte_01; 02	2	Atlantic sharpnose shark	*Prenhoca; milho verde*	AY830763-64
	*Carcharhinus signatus*	Csi_01	1	Night shark	*-*	AY830744
	*Carcharhinus perezii*	Cpe_01; 02	2	Caribbean reef shark	*Fidalgo; azul*	AY830739-40
	*Carcharhinus longimanus*	Clo_01; 02	2	Oceanic whitetip shark	-	AY830735-36
	*Carcharhinus obscurus*	Cob_01; 02	2	Dusky shark	*Fidalgo*	AY830737-38
	*Carcharhinus falciformis*	Cfa_01; 02	2	Silky shark	*Lombo-preto*	AY830725-26
	*Carcharhinus plumbeus*	Cpl_01; 02	2	Sandbar shark	*Cação-galhudo*	AY830741-42
	*Carcharhinus altimus*	Cal_01	1	Bignose shark	-	AY830722
	*Carcharhinus acronotus*	Cac_01	1	Blacknose shark	*Flamengo*	AY830721
	*Carcharhinus brevipinna*	Cbr_01	1	Spinner shark	-	AY830723
	*Carcharhinus limbatus*	Cli_01; 02	2	Blacktip shark	*Sacurí; galha preta*	AY830731-32
	*Carcharhinus limbatus*	Cli_293	1	-	*Sacurí; galha preta*	Present study FJ598682
	*Carcharhinus porosus*	Cpo_01	1	Smalltail shark	*Prenhoca*	AY830743
	*Carcharhinus porosus*	Cpo_288	1	-	*Prenhoca*	Present study FJ598683
	*Carcharhinus leucas*	Cle_01	1	Bull shark	*Boca redonda*	AY830730
	*Carcharhinus leucas*	Cle_292	1	-	*Boca redonda*	Present study FJ598691
	*Carcharhinus isodon*	Cis_01; 02	2	Finetooth shark	-	AY830728-29
	*Isogomphodon oxyrhyncus*	Iox_291	1	Daggernose shark	*Cação pato*	Present study FJ598693
	*Prionacea glauca*	Pgl_01; 02	2	Blue shark	-	AY830761-62
	*Negaprion brevirostris*	Nbr_01	1	Lemon shark	*Cação areia*	AY830756
	*Galeocerdo cuvier*	Gcu_01	1	Tiger shark	*Tintureira; jaguará*	AY830746

Triakidae	Mustelus norrisi	Mno_01	1	Narrowfin smooth-hound	-	AY830755
	*Mustelus canis*	Mca_01	1	Dusky smooth-hound	*Canejo*	AY830754

Alopidae	*Alopias superciliosus*^d^	Asu_01; 02	2	Bigeye thresher	-	AY830718-19
	*Alopias vulpinus*^d^	Avu_01	1	Thresher	-	AY830720

a - (Szpilman, 2004); b - (Elias MP, MSc Dissertation, Universidade Federal do Pará, Belém-PA, 2004); c - GenBank accession number (Greig *et al.*, 2005); d - Used only for the genetic divergence analysis.

**Table 2:** Number of samples collected with their respective haplotypes and GenBank access.

Haplotypes	N	GenBank
Hap_01	4	FJ598677
Hap_02	3	FJ598675
Hap_03	1	FJ598676
Hap_04	8	FJ598670
Hap_05	13	FJ598668
Hap_06	1	FJ598667
Hap_07	2	FJ598666
Hap_08	6	FJ598671
Hap_09	1	FJ598672
Hap_10	4	FJ598669
Hap_11	4	FJ598678
Hap_12	2	FJ598679
Hap_13	1	FJ598681
Hap_14	1	FJ598680
Hap_15	1	FJ598692
Hap_16	1	FJ598684
Hap_17	1	FJ598685
Hap_18	33	FJ598686
Hap_19	1	FJ598689
Hap_20	1	FJ598688
Hap_21	7	FJ598690
Hap_22	12	FJ598687
Hap_23	1	FJ598673
Hap_24	1	FJ598674
Hap_25	1	FJ598694
Hap_26	2	FJ598665
Hap_27	2	FJ598660
Hap_28	1	FJ598661
Hap_29	6	FJ598664

**Table 3:** Uncorrected “p” distance (%) between identified species and those from Table 1. Values are the medium between each comparison. a – sequences from Greig *et al.* (2005); b – voucher specimens collected during the present study; () number of specimens; - Not calculated.

	*Sle*^*a*^	*Ssp.*^*b*^	*Sti*^*ab*^	*Stu*^*b*^	*Smo*^*ab*^	*Rhz*^*ab*^	*Cpe*^*ab*^	*Cfa*^*ab*^	*Cpl*^*ab*^	*Cal*^*a*^	*Cac*^*ab*^	*Cli*^*ab*^	*Cpo*^*ab*^	*Cle*^*ab*^	*Iox*^*b*^	*Gcu*^*ab*^	*Mno*^*a*^	*Mca*^*a*^	*Asu*^*a*^	*Avu*^*a*^
*Sle*^*a*^(1)	-																			
*Ssp.*^*b*^(3)	2.5	0																		
*Sti*^*ab*^(3)	3.3	3.6	0.4																	
*Stu*^*b*^(8)	4.3	4.7	2.4	0.06																
*Smo*^*ab*^(3)	5.6	5.9	5.5	5.6	0															
*Rhz*^*ab*^(37)	8.3	7.9	7.7	7.66	7.67	0.4														
*Cpe*^*ab*^(4)	9	9.2	8.75	8.9	9.77	6.8	0.18													
*Cfa*^*ab*^(6)	9.05	9.25	8.6	8.48	9.3	6.32	3.85	0.1												
*Cpl*^*ab*^(4)	8.6	8.9	8.97	8.46	8.73	6.52	4.57	3	0.2											
*Cal*^*a*^(1)	8.6	9	8.83	8.43	8.6	6.58	4.42	3.05	0.53	-										
*Cac*^*ab*^(9)	8.6	8.7	8.48	8.8	8.85	6.56	4.32	3.35	3.88	3.94	0.14									
*Cli*^*ab*^(3)	8.6	9.1	8.4	8.7	9.56	7.37	4.85	4.23	5	4.73	4.36	0.06								
*Cpo*^*ab*^(58)	9.1	9.04	8.96	9	9.28	7	4.89	4.53	5.41	5.15	4.08	5.69	0.32							
*Cle*^*ab*^(3)	9.4	10	8.83	8.88	9.33	7.3	4.22	3.7	4.45	4.13	3.66	4.95	4.99	0.13						
*Iox*^*b*^(1)	8.7	9	8.2	8.3	8.4	6.5	4.82	4.15	4.53	4.2	3.8	4.6	4.48	4.13	-					
*Gcu*^*ab*^(2)	9.3	9.6	8.33	8.46	9.1	8.12	8.27	8.15	8.46	8.5	7.56	8.33	8.03	8.63	7.7	0				
*Mno*^*a*^(1)	9.8	10.8	10.4	10.46	9.7	9.26	8.35	7.45	8.56	8.6	8.2	7.96	7.67	8.06	8.2	9.8	-			
*Mca*^*a*^(1)	9.6	10.3	9.63	10.13	10.1	8.78	7.42	6.65	7.83	7.5	7.34	7.3	7.27	6.96	8	9	3.1	-		
*Asu*^*a*^(2)	15	15	14.9	14.23	14.2	14.78	15	15.35	14	14	14.44	14.16	14.72	15.16	14.7	14.7	13	12.3	0	
*Avu*^*a*^(1)	14.5	14.7	14.63	14	13.9	14.2	14.77	14.95	13.4	13.4	13.96	14	14.32	14.73	14.6	13.7	13	12.9	5.5	-

**Table 4:** Matrix comparing the name given to the specimen by the supplier and species identified through molecular analysis of the 122 samples of shark tissue collected during the present study.

Name given by supplier	N	Number of specimens identified as:												
		Cfa	Cpl/Cal	Cpo	Cac	Cpe	Cle	Rhz	Gcu	Smo	Stu	Sle	Sti	Ssp
*Sacurí*	9	1	4	1	-	-	-	1	-	-	1	-	-	1
*Lombo preto*	4	3	-	-	-	-	-	1	-	-	-	-	-	-
*Milho verde*	84	-	-	48	5	-	-	29	-	1	-	-	1	-
*Flamengo*	1	-	-	-	-	-	-	1	-	-	-	-	-	-
*Cação areia*	9	-	-	2	2	-	-	1	-	-	4	-	-	-
*Pana*	2	-	-	-	-	-	1	-	-	1	-	-	-	-
*Maxoté*	1	-	-	-	-	1	-	-	-	-	-	-	-	-
*Cação pato*	1	-	-	-	-	-	-	-	1	-	-	-	-	-
*Tubarão branco*	1	-	-	-	-	-	-	-	-	-	-	-	-	1
No name given	10	-	-	6	-	1	-	2	-	-	1	-	-	-

N – Number of specimens identified.

## References

[Abercrombieetal2005] Abercrombie D.L., Clarke S.C., Shivji M.S. (2005). Global-scale genetic identification of hammerhead sharks: Application to assessment of the international fin trade and law enforcement. Conserv Genet.

[Baumetal2003] Baum J.K., Myers R.A., Kehler D.G., Worm B., Harley S.J., Doherty P.A. (2003). Collapse and conservation of shark populations in the northwest Atlantic. Science.

[BaumandMyers2004] Baum J.K., Myers R.A. (2004). Shifting baselines and the decline of pelagic sharks in the Gulf of Mexico. Ecol Lett.

[Camhietal1998] Camhi M., Fowler S., Musick J., Bräutigan F.S. (1998). Sharks & their relatives: Ecology and Conservation. IUCN/SSC Shark Specialist Group.

[Castro1993] Castro J.I. (1993). A Field Guide to the Sharks Commonly Caught in Commercial Fisheries of the Southeastern United States: NOAA Technical Memorandum NMFS-SEFSC-338.

[Clarkeetal2006a] Clarke S.C., Magnussen J.E., Abercrombie D.L., Mcallister M.D., Shivji M.S. (2006a). Identification of shark species composition and proportion in the Hong Kong shark fin market based on molecular genetics and trade records. Conserv Biol.

[Clarkeetal2006b] Clarke S.C., McAllister M.K., Milner-Gulland E.J., Kirkwood G.P., Michielsens C.G.J., Agnew D.J., Pikitch E.K., Nakano H., Shivji M.S. (2006b). Global estimates of shark catches using trade records from commercial markets. Ecol Lett.

[Chanetal2003] Chan R.W.K., Dixon P.I., Pepperell J.G.D., Reid D. (2003). Application of DNA-based techniques for the identification of whaler sharks (*Carcharhinus* spp) caught in protective beach meshing and by recreational fisheries off the coast of New South Wales. Fish Bull (Wash D C).

[CoelhoandErzini2008] Coelho R., Erzini K. (2008). Identification of deep water lantern sharks (Chondrichthyes, Etmopteridae) using morphometric data and multivariate analysis. J Mar Biol Assoc UK.

[Compagno1984] Compagno L.J.V. (1984). FAO species catalogue. v. 4. Sharks of the world. An annotated and illustrated catalogue of shark species known to date. Part 2. Carcharhiniformes. FAO Fish Synop.

[Cortes2002] Cortés E. (2002). Stock assessment of small coastal sharks in the U. S. Atlantic and Gulf of Mexico. Conserv Biol.

[Douadyetal2003] Douady C.J., Dosay M., Shivji M.S., Stanhope M.J. (2003). Molecular phylogenetic evidence refuting the hypothesis of Batoidea (rays and skates) as derived sharks. Mol Phylogenet Evol.

[Dulvyetal2003] Dulvy N.K., Sadovy Y., Reynolds J.D. (2003). Extinction vulnerability in marine populations. Fish Fish.

[Fundacao2006] Fundação P.R.O.Z.E.E. (2006). Relatório Final do Projeto de Monitoramento da Atividade Pesqueira no Litoral do Brasil – Projeto Estatpesca.

[FAO2000] FAO (FoodAgriculture Organization) (2000). Conservation and Management of Sharks. FAO Tech Guide Resp Fish.

[Greigetal2005] Greig T.W., Moore M.K., Woodley C.M., Quattro J.M. (2005). Mitochondrial gene sequences useful for species identification of western North Atlantic Ocean sharks. Fish Bull (Wash D C).

[Hall1999] Hall T.A. (1999). BioEdit: A user-friendly biological sequence alignment editor and analysis program for Windows 95/98/NT. Nucleic Acids Symp Ser.

[HeistandGold1999] Heist E.J., Gold J.R. (1999). Genetic identification of sharks in the U. S. Atlantic large coastal shark fishery. Fish Bull (Wash D C).

[Hoelzel2001] Hoelzel A.R. (2001). Shark fishing in fin soup. Conserv Genet.

[HoenigandGruber1990] Hoenig J.M., Gruber S.H., Pratt H.L., Gruber S.H. (1990). Life-history patterns in the elasmobranchs: Implications for fisheries management. Elasmobranchs as Living Resources: Advances in the Biology, Ecology, Systematics, and the Status of the Fisheries.  Proceedings of the Second United States-Japan Workshop East-West Center.

[Iglesiasetal2005] Iglésias S.P., Lecointre G., Sellos D.Y. (2005). Extensive paraphylies within sharks of the order Carcharhiniformes inferred from nuclear and mitochondrial genes. Mol Phylogenet Evol.

[LessaandSantana1998] Lessa R., Santana F.M. (1998). Age determination and growth of the smalltail shark, *Carcharhinus porosus*, from northern Brazil. Mar Freshw Res.

[Magnussenetal2007] Magnussen J.E., Pikitch E.K., Clarke S.C., Nicholson C., Hoelzel A.R., Shivji M.S. (2007). Genetic tracking of basking shark products in international trade. Anim Conserv.

[Myersetal2007] Myers R.A., Baum J.K., Shepherd T.D., Powers S.P., Peterson C.H. (2007). Cascading effects of the loss of apex predatory sharks from a coastal ocean. Science.

[Paiva1997] Paiva M.P. (1997). Recursos Pesqueiros Estuarinos e Marinhos do Brasil.

[Panketal2001] Pank M., Stanhope M., Natanson L., Kohler N., Shivji M. (2001). Rapid and simultaneous identification of body parts from the morphologically similar sharks *Carcharhinus obscurus* and *Carcharhinus plumbeus* (Carcharhinidae) using multiplex PCR. Mar Biotechnol (NY).

[Pinhaletal2008] Pinhal D., Gadig O.B.F., Wasko A.P., Oliveira C., Ron E., Foresti F., Martins C. (2008). Discrimination of Shark species by simple PCR of 5S rDNA repeats. Genet Mol Biol.

[Quattroetal2006] Quattro J.M., Stone D.S., Driggers W.B., Anderson C.A., Priede K.A., Hoppmann E.C., Campbell N.H., Duncan K.M., Grady J.M. (2006). Genetic evidence of cryptic speciation within hammerhead sharks (Genus *Sphyrna*). Mar Biol.

[RambautandDrummond2004] Rambaut A., Drummond A.J. (2004). Tracer, v. 1.1.

[RonquistandHuelsenbeck2003] Ronquist F., Huelsenbeck J.P. (2003). MrBayes 3: Bayesian inference of phylogenetic trees. Bioinformatics.

[Sambrooketal1989] Sambrook J., Fritsch E.F., Maniatis T. (1989). Molecular Cloning: A Laboratory Manual.

[Sangeretal1977] Sanger F., Nichlen S., Coulson A.R. (1977). DNA sequencing with chain-termination inhibitors. Proc Natl Acad Sci USA.

[Schneider2007] Schneider H. (2007). Métodos de Análise Filogenética: Um Guia Prático.

[ShepherdandMyers2005] Shepherd T.D., Myers R.A. (2005). Direct and indirect fishery effects on small coastal elasmobranchs in the northern Gulf of Mexico. Ecol Lett.

[Shivjietal2002] Shivji M.S., Clarke S., Pank M., Natanson L., Kohler N., Stanhope M. (2002). Genetic identification of pelagic shark body parts for conservation and trade monitoring. Conserv Biol.

[Shivjietal2005] Shivji M.S., Chapman D.D., Pikitch E.K., Raymond P.W. (2005). Genetic profiling reveals illegal international trade in fins of the great white shark, *Carcharodon carcharias*. Conserv Genet.

[StevensandWayte1998] Stevens J.D., Wayte S.E. (1998). A review of Australia's pelagic shark resources: FRDC Project n. 98/107.

[Stevensetal2000] Stevens J.D., Bonfil R., Dulvy N.K., Walker P.A. (2000). The effects of fishing on sharks, rays, and chimaeras (Chondrichthyans) and the implications for marine ecosystems. ICES J Mar Sci.

[Szpilman2004] Szpilman M. (2004). Tubarões no Brasil: Guia Prático de Identificação.

[Thompsonetal1997] Thompson J.D., Gibson T.J., Plewniak F., Jeanmougin J., Higgins D.G. (1997). The clustal- windows interface: Flexible strategies for multiple sequence alignment aided by quality analysis tools. Nucleic Acids Res.

[Valenzuelaetal2008] Valenzuela A., Bustamante C., Lamilla J. (2008). Morphological characteristics of five bycatch sharks caught by southern Chilean demersal longline fisheries. Sci Mar.

[XiaandXie2001] Xia X., Xie Z. (2001). DAMBE: Data analysis in molecular biology and evolution. J Hered.

[Ward2000] Ward R.D. (2000). Genetics in fisheries management. Hydrobiologia.

[Wardetal2008] Ward R.D., Holmes B.H., William T.W., Last P.R. (2008). DNA barcoding Australasian chondrichthyans: Results and potential uses in conservation. Mar Freshw Res.

